# The histidine-rich peptide LAH4-L1 strongly promotes PAMAM-mediated transfection at low nitrogen to phosphorus ratios in the presence of serum

**DOI:** 10.1038/s41598-017-10049-y

**Published:** 2017-08-29

**Authors:** Nan Liu, Burkhard Bechinger, Regine Süss

**Affiliations:** 1 Institute of Pharmaceutical Sciences, Department of Pharmaceutical Technology and Biopharmacy and Freiburger Materialforschungszentrum (FMF), Albert Ludwig University Freiburg, Sonnenstr. 5, 79104 Freiburg, Germany; 2 University of Strasbourg/CNRS, Membrane Biophysics and NMR, Chemistry Institute UMR7177, rue Blaise Pascal 1, 67008 Strasbourg, France

## Abstract

Non-viral vectors are widely used and investigated for the delivery of genetic material into cells. However, gene delivery barriers like lysosomal degradation, serum inhibition and transient gene expression so far still limit their clinical applications. Aiming to overcome these limitations, a pH-sensitive hybrid gene vector (PSL complex) was designed by self-assembly of poly(amidoamine) (PAMAM) dendrimers, the histidine-rich peptide LAH4-L1 and the sleeping beauty transposon system (SB transposon system, a plasmid system capable of efficient and precise genomic insertion). Transfection studies revealed that PSL complexes achieved excellent efficiency in all investigated cell lines (higher than 90% in HeLa cells and over 30% in MDCK cells, a difficult-to-transfect cell line). Additionally, the PSL complexes showed high serum tolerance and exhibited outstanding transfection efficiency even in medium containing 50% serum (higher than 90% in HeLa cells). Moreover, a high level of long-term gene expression (over 30% in HeLa cells) was observed. Furthermore, PSL complexes not only resulted in high endocytosis, but also showed enhanced ability of endosomal escape compared to PAMAM/DNA complexes. These results demonstrate that simple association of PAMAM dendrimers, LAH4-L1 peptides and the SB transposon system by self-assembly is a general and promising strategy for efficient and safe gene delivery.

## Introduction

Gene therapy is a promising therapeutic strategy for diseases caused by genetic defects and, therefore, a variety of gene delivery systems have been developed^1, 2^. Non-viral gene delivery systems are widely used, as they are relatively safe and easy to synthesize with lower immunogenicity and larger genetic payloads^3^. Among the non-viral vectors, poly(amidoamine) (PAMAM) dendrimers, cell penetrating peptides and the sleeping beauty (SB) transposon system are three typical components of non-viral vectors with different strategies to overcome barriers to transfection, however, each of them suffers intrinsic drawbacks^4–6^.

PAMAM dendrimer possess a unique surface of primary amino groups which associate plasmid DNA or siRNA^7^ via electrostatic interaction, resulting in the formation of nanoscaled complexes, which facilitate cellular uptake and aid in the endosomal escape by the “proton sponge effect”^8^. However, this requires the formation of complexes at high nitrogen to phosphorus ratios (N/P ratios, *i.e*. the ratio of the number of terminal amine groups on the PAMAM dendrimer to the number of phosphate groups in the nucleic acids), leading to relatively high cytotoxicity as well^9^. Besides, PAMAM dendrimer-mediated transfection is less effective in the presence of serum^10^.

LAH4-L1 is a short cationic histidine-rich amphipathic peptide from the LAH4 peptide family. The primary sequence of LAH4-L1 (KKALLAHALHLLALLALHLAHALKKA) results in an amphipathic helix conformation when complexes are formed with DNA or siRNA as well as when bound to membranes^11^. This amphipathic peptide contains four hydrophilic histidine residues and a mixture of hydrophobic residues (mainly leucine and alanine residues). Therefore, the LAH4-L1 peptide is positively charged at physiologic pH due to the hydrophilic surface in aqueous solution, which is a favor for binding and condensation of nucleic acids, whereas its hydrophobic surface is suitable for efficient membrane interactions^12, 13^. Similar to cationic PAMAM dendrimers, the LAH4-L1 peptide spontaneously forms complexes with DNA or siRNA but uses a different strategy for endosomal escape. It promotes endosomal escape by “endosomal destabilization effect” owing to its ability to destabilize the endosomal membrane when the four histidine residues are positively charged at low pH^14, 15^. Although LAH4-L1 peptide mediated transfection is less cytotoxic and can be performed in the presence of serum, it provides relatively low DNA transfection efficiency.

The SB transposon system (SBTS) is a plasmid system consisting of a SB transposase and a SB transposon that enables precise integration of a gene of interest into the genomes of cells by a cut-and-paste process. Briefly, the SB transposon is a Tc1/mariner-like transposon that consists of a gene of interest flanked by terminal inverted repeats (IRs). During the transposition, SB transposases bind to the four recognition sequences in IRs and excise the transposon containing the gene of interest from the delivered plasmid DNA. The transposase possesses the NLS that promotes the nuclear import. The pre-integration complex consists of transposons flanked by IRs and transposases binding to IRs. The transposases facilitate the integration of the transposon into the targeted TA-dinucleotides of the chromosome. Thus, the gene of interest is stably integrated into the host genome, leading to long-term exogenous gene expression^16, 17^. Therefore, as alternatives to viral vectors, SBTS can induce long-term expression of a therapeutic gene with less safety concerns, expending the scientific and clinical applications of non-viral gene vectors. However, the SBTS requires a delivery system to effectively enter a cell^18, 19^.

In this work, aiming to overcome the limitations of non-viral vectors mentioned above, we hypothesized that the cationic PAMAM dendrimers and LAH4-L1 peptides could associate with negatively charged SBTS by electrostatic interaction, thus forming PAMAM/SBTS/LAH4-L1 (PSL) complexes. Therefore, the PSL complexes could combine two strategies for endosomal escape and the capability of long-term gene expression. The SBTS contains a GFP reporter gene and effective gene delivery of PSL complexes was analyzed by flow cytometry and fluorescence microscopy. The presented data show that PSL complexes resulted in high-level of transient and long-term gene expression without cytotoxicity. Besides, PSL complexes showed excellent serum tolerance for all investigated cell lines. These results demonstrate that PSL complexes are promising gene vectors for highly efficient and safe transfection.

## Results

### Characterization of Sleeping beauty transposon system/LAH4-L1 (SL) complexes, PAMAM/Sleeping beauty transposon system/LAH4-L1 (PSL) complexes and PAMAM/Sleeping beauty transposon system (PS) complexes

As expected, PAMAM, LAH4-L1 and DNA effectively form positively charged nanoparticles at the N/P ratio of 5 (Supplementary Figures 1 and 2). As shown in Supplementary Figure 1, the particle size of SL complexes formed by combination of DNA plasmid with LAH4-L1 peptide at a weight ratio of 1/1 was 135 nm, which was larger than the particle size of PSL complexes (60 nm) and the particle size of PS complexes (65 nm). However, the values of PDI were higher than 0.1, the particle sizes of these complexes revealed high dispersity.

Regarding the zeta-potential (Supplementary Figure 2), SL complexes showed a negative zeta potential when the weight ratio of LAH4-L1 to SBTS was 1. Addition of PAMAM reversed surface charge from negative to positive, suggesting that PAMAM could bind to the surface of SBTS/LAH4-L1 complexes.

To evaluate the formation of the complexes, agarose gel electrophoresis of SL complexes, PSL complexes and PS complexes was performed. As shown in Supplementary Figure 3a, the mobility of DNA in SL complexes was completely retarded when the weight ratio of LAH4-L1 to SBTS was 3, suggesting that SL complexes were formed at this ratio. As shown in Supplementary Figure 3b, electrophoretic mobility of DNA in PSL complexes was completely retarded when the weight ratio of PAMAM to DNA was higher than 1, suggesting that PSL complexes were formed when the PAMAM/DNA weight ratio was higher than 1. As shown in Supplementary Figure 3c, similar electrophoretic mobility of DNA was observed compared to Supplementary Figure 3b, suggesting that PS complexes were formed when the PAMAM/DNA weight ratio was higher than 1.

### Comparison of transfection efficiency of PAMAM/SBTS (PS) complexes with PAMAM/SBTS/LAH4-L1 (PSL) complexes

**Figure 1:**
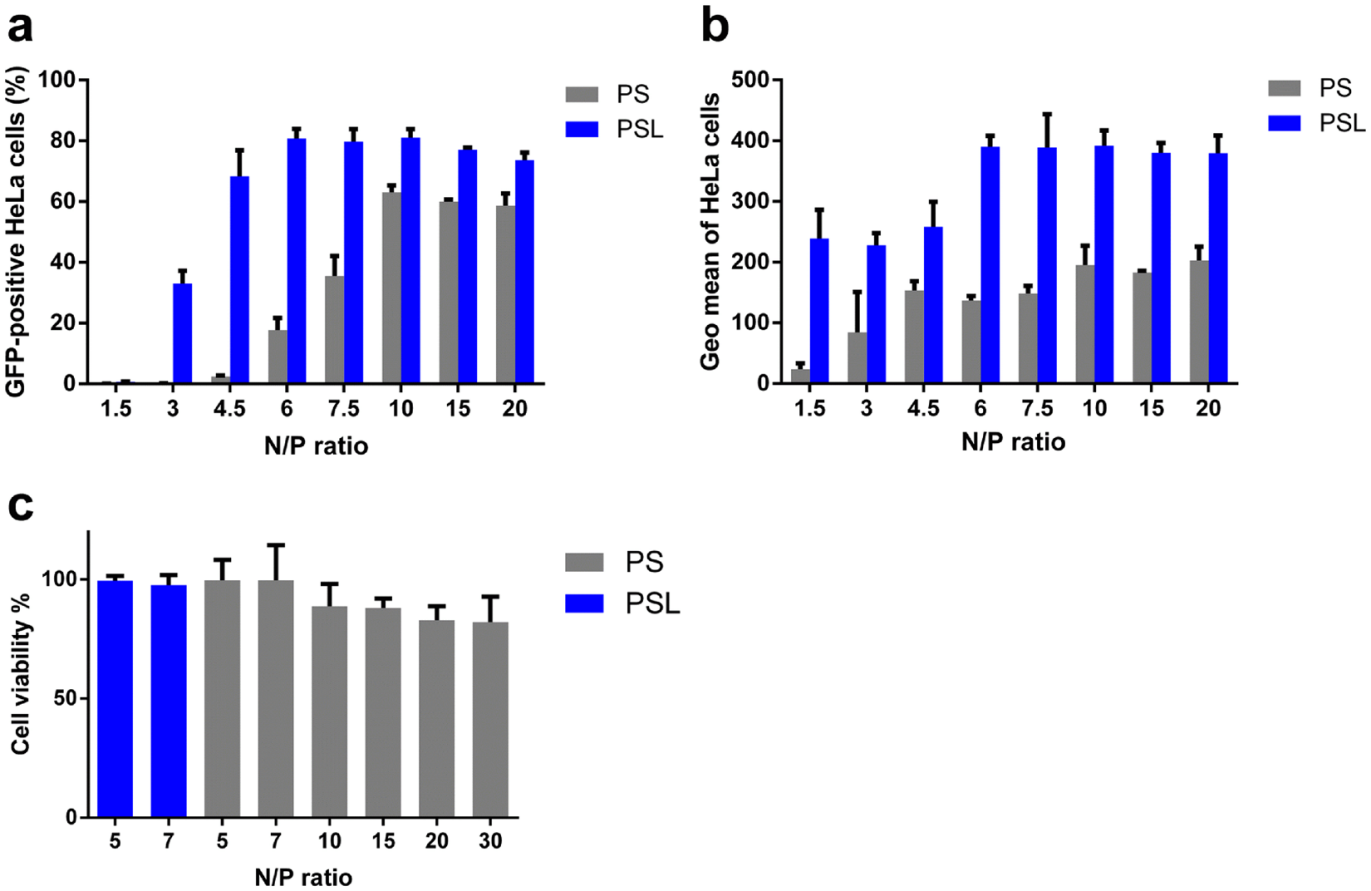
Transfection efficiency and cytotoxicity of PSL and PS complexes in HeLa cells at various N/P ratios in the presence of 10% FCS. Transfection was performed at a dose of 0.3 μg of DNA per well in a total volume of 200 μl. GFP expression was quantified by flow cytometry after 24 hours. (**a**) Percentage of GFP-positive HeLa cells. (**b**) Geometric mean of fluorescence intensity of GFP-positive cells. (**c**) Cell viability assay of HeLa cells after transfection. Data represent mean ± standard deviation of 3 independent experiments.

To investigate, whether incorporation of LAH4-L1 peptide into PAMAM based gene delivery systems enhances transfection, HeLa cells were transfected with PS and PSL complexes prepared at N/P ratio from 1.5 to 20 (Tables 1 and 2) and gene transfection efficiency was analyzed by flow cytometry. As shown in Fig. 1a, transfection efficiency of PSL was significantly higher than that of PS at all investigated N/P ratios, especially at low N/P ratio. At an N/P ratio of 4.5, transfection efficiency of PSL complexes was 68%, whereas PS complexes showed extremely poor transfection efficiency. Moreover, the geometric mean of fluorescence intensity of GFP-positive cells in the PSL group exhibited more than a 2-fold increase than that in PS group at all investigated N/P ratios (Fig. 1b), suggesting that PSL complexes could increase the expression level of GFP per GFP-positive cell compared to PS complexes.

**Table 1:** Details of the contents mixed to form self assembled PS complexes at various N/P ratios in Fig. 1.

N/P ratio	PAMAM/DNA, w/w
1.5	1.02/1
3	2.04/1
4.5	3.06/1
6	4.08/1
7.5	5.1/1
10	6.8/1
15	10.2/1
20	13.6/1

**Table 2:** Details of the contents mixed to form self assembled PSL complexes at various N/P ratios in Fig. 1.

N/P ratio	PAMAM/DNA/LAH4-L1, w/w/w
1.5	1.02/1/1
3	2.04/1/1
4.5	3.06/1/1
6	4.08/1/1
7.5	5.1/1/1
10	6.8/1/1
15	10.2/1/1
20	13.6/1/1

It has been shown that cationic non-viral gene vectors encounter the efficiency-cytotoxicity dilemma^9^. To test the cytotoxicity of PS and PSL complexes, the cytotoxicity were evaluated in HeLa cells by CellTiter-Glo^®^ Luminescent Cell Viability Assay after transfection. As shown in Fig. 1c, PAMAM based gene delivery system induced an N/P ratio-dependent increment of cytotoxicity in HeLa cells. The cytotoxicity of PS and PSL complexes was relatively low (cell viability > 90%) when the N/P ratio is less than 7. However, cell viability dropped slightly when the N/P ratio increased up to 30. It can be concluded, that PSL exhibit very high transfection efficiency at low N/P ratio combined with very low cytotoxicity, thereby overcoming the efficiency-cytotoxicity dilemma of cationic non-viral gene vectors.

### Comparison of transfection efficiency of SBTS/LAH4-L1 (SL) complexes with PSL complexes

The LAH4-L1 peptide has been reported to function as vector for efficient delivery of DNA or siRNA owing to its ability to destabilize the endosomal membrane^12, 14^. To further investigate whether the enhanced transfection efficiency induced by PSL complexes at low N/P ratio only benefits from the DNA transfection capability of LAH4-L1 peptide, transfection efficiency of SL complexes was tested at various weight ratios of peptide to DNA from 1 to 6 (Table 3). As a comparison, PSL complexes with indicated LAH4-L1/DNA weight ratio were prepared and investigated at an N/P ratio of 5 (Table 4).

**Table 3:** Details of the contents mixed to form self assembled SL complexes prepared at various DNA/LAH4-L1 weight ratios in Fig. 2.

LAH4-L1/DNA weight ratio	LAH4-L1/DNA, w/w
1	1/1
2	2/1
3	3/1
4	4/1
5	5/1
6	6/1

**Table 4:** Details of the contents mixed to form self assembled PSL complexes (N/P 5) prepared at various DNA/LAH4-L1 weight ratios in Fig. 2.

LAH4-L1/DNA weight ratio	PAMAM/DNA/LAH4-L1, w/w/w
0.25	3.4/1/0.25
0.5	3.4/1/0.5
0.75	3.4/1/0.75
1	3.4/1/1
2	3.4/1/2
3	3.4/1/3
4	3.4/1/4

**Figure 2:**
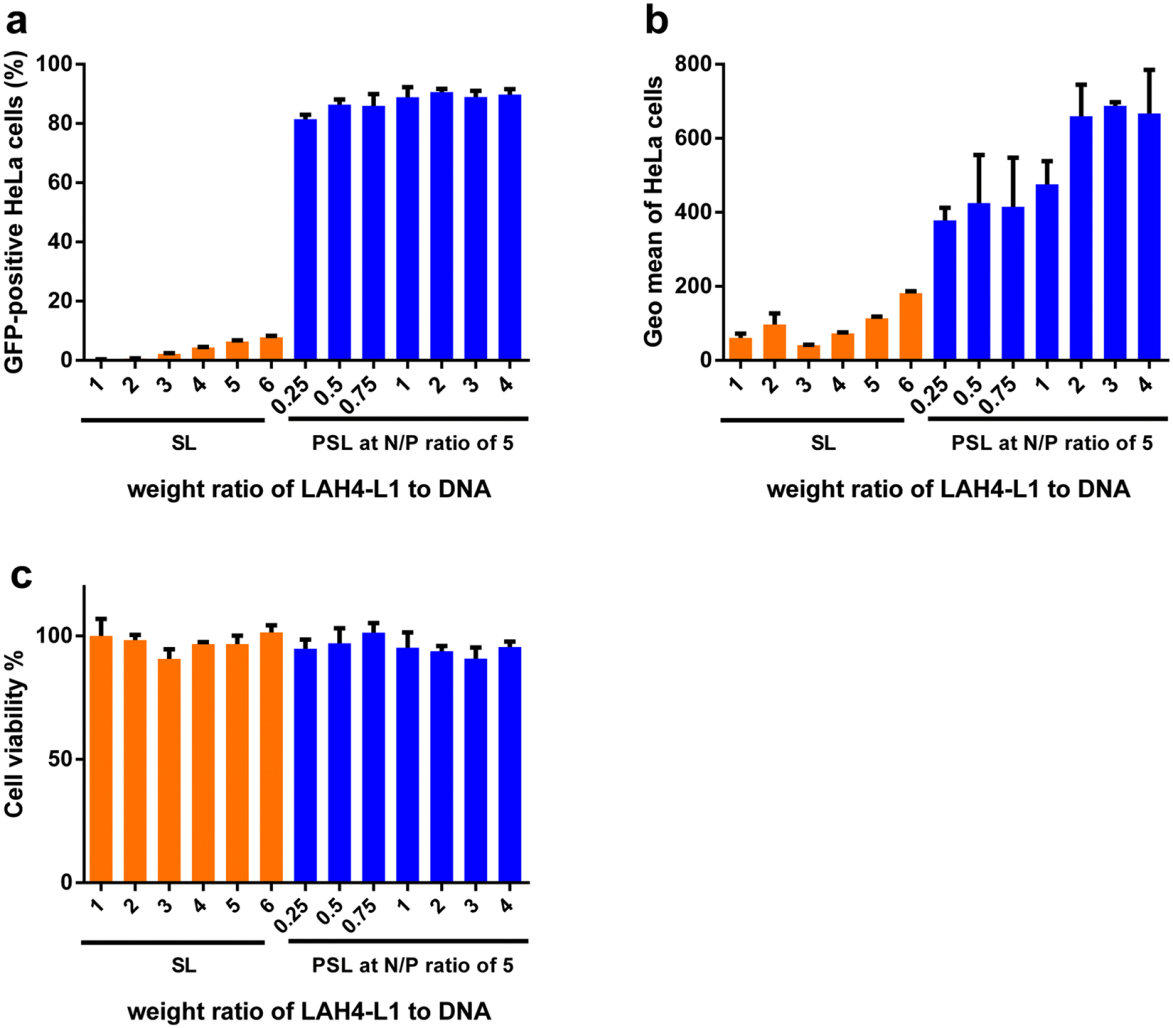
Transfection efficiency and cytotoxicity of SL complexes and PSL complexes in HeLa cells at various LAH4-L1/DNA weight ratios in the presence of 10% FCS. Transfection was performed at a dose of 0.3 μg of DNA per well in a total volume of 200 μl. (**a**) Percentage of GFP-positive HeLa cells. (**b**) Geometric mean of fluorescence intensity of GFP-positive cells. (**c**) Cell viability assay of HeLa cells after transfection. Data represent mean ± standard deviation of 3 independent experiments.

As shown in Fig. 2a,b, the LAH4-L1 peptide alone induced very low transfection efficiency even after dramatically increasing the amount of peptide added. However, the PSL complexes could successfully transfer GFP gene into HeLa cells with less amount of peptide added. Compared to SL complexes (8% at LAH4-L1/DNA weight ratio of 6), PSL complexes could achieve higher than 80% transfection efficiency even at a very low LAH4-L1/DNA weight ratio of 0.25 in HeLa cells. In addition, the geometric mean of GFP-positive cells in the PSL group was higher than that in the PS group and exhibited a peptide-dose-dependent enhancement. This indicated that the high efficiency of PSL complexes at low N/P ratio benefited not only from LAH4-L1 but also from the cationic PAMAM polymer. As shown in Fig. 2c, the cytotoxicity of SL complexes and PSL complexes was relatively low (cell viability > 90%).

To further investigate the multifaceted cell death pathways caused by PSL complexes, PS complexes and SL complexes, the viable, early apoptotic, late apoptotic and necrotic cells were detected in HeLa cells by Annexin-V FITC apoptosis detection kit. As shown in supplemental Fig. 4, necrosis and apoptosis caused by PSL complexes at the N/P ratio of 5, PS complexes at the N/P ratio of 5 or SL complexes at the DNA/LAH4-L1 weight ratio of 1 were similar to those in the control group. PS complexes prepared at the N/P ratio of 20 caused the highest level of apoptosis (6.8%).

To balance the efficiency and cytotoxicity, the N/P ratio of 5 and LAH4-L1/DNA weight ratio of 1 were used for the preparation of PSL complexes and PS complexes in the subsequent studies.

### Endosomal escape abilities of PS and PSL complexes

Inefficient endosomal escape is considered as one of the major barriers to successful transfection of gene vectors^15^. It has been previously demonstrated that LAH4-L1 peptide can effectively disrupt the endosomal membrane upon endosomal acidification^13, 20^. To investigate, whether LAH4-L1 could promote endosomal escape of DNA delivered by PSL complexes, plasmid DNA was labeled with YOYO^®^-1 (green) and late endosome/lysosome was stained with Lysotracker Red (red) for the investigation of intracellular distribution. The colocalization of plasmid DNA with lysosomal markers is represented in yellow in merged images.

**Figure 3:**
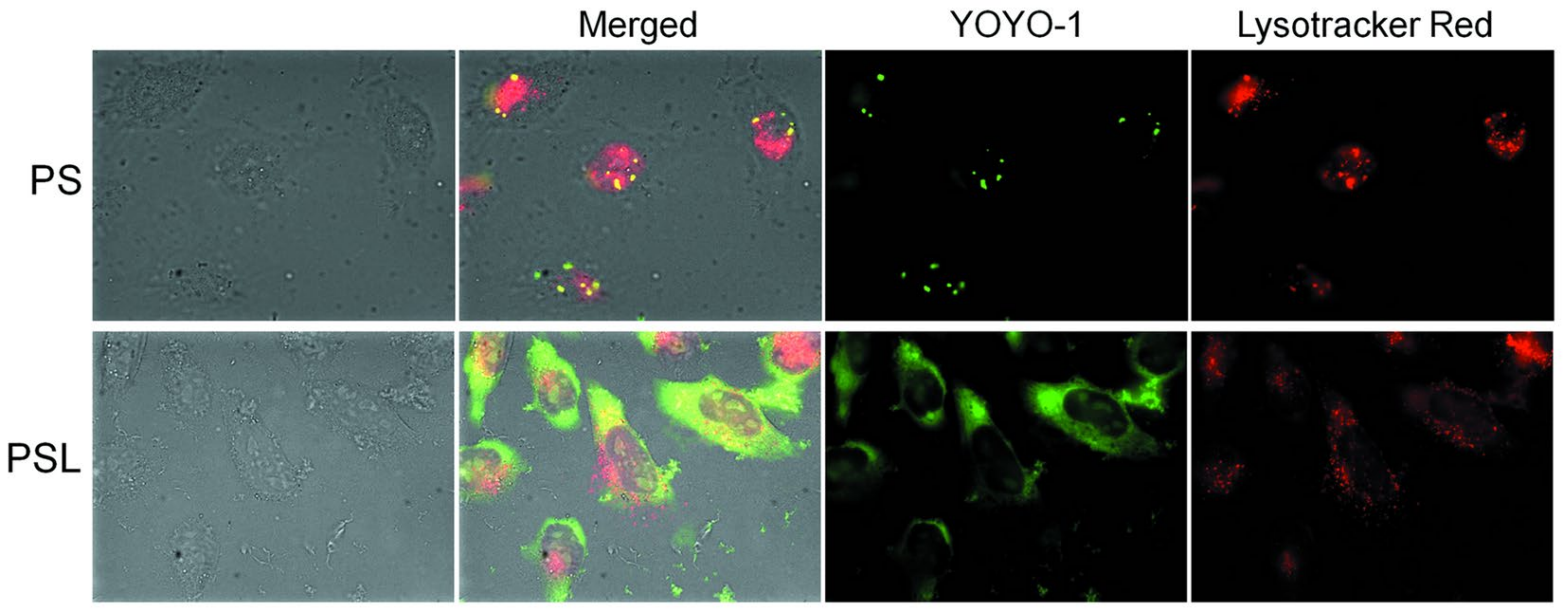
Cellular distribution of PS and PSL complexes (YOYO®-1 labeled, green) in HeLa cells at 4 h post transfection under fluorescence microscope at a magnification of 630-fold. Late endosome/lysosome was stained with Lysotracker Red DND-99 (red). The colocalization of PS or PSL complexes with lysosomes is represented in yellow in merged images.

As shown in Fig. 3, both PS and PSL complexes induced efficient cellular uptake of DNA. For PS complexes, most of the DNA was colocalized with the lysosomal marker, suggesting that endosomal escape of DNA delivered by PS complexes was inefficient leading to low transfection efficiency. In contrast, for DNA delivered by PSL complexes, the micrographs clearly show, that DNA was released into the cytoplasm and only few fluorescence was found inside the lysosomal vesicles, suggesting that the presence of LAH4-L1 peptide in the PSL complexes facilitated the endosomal escape of DNA from PSL complexes, thus resulting in very high transfection efficiency.

**Figure 4:**
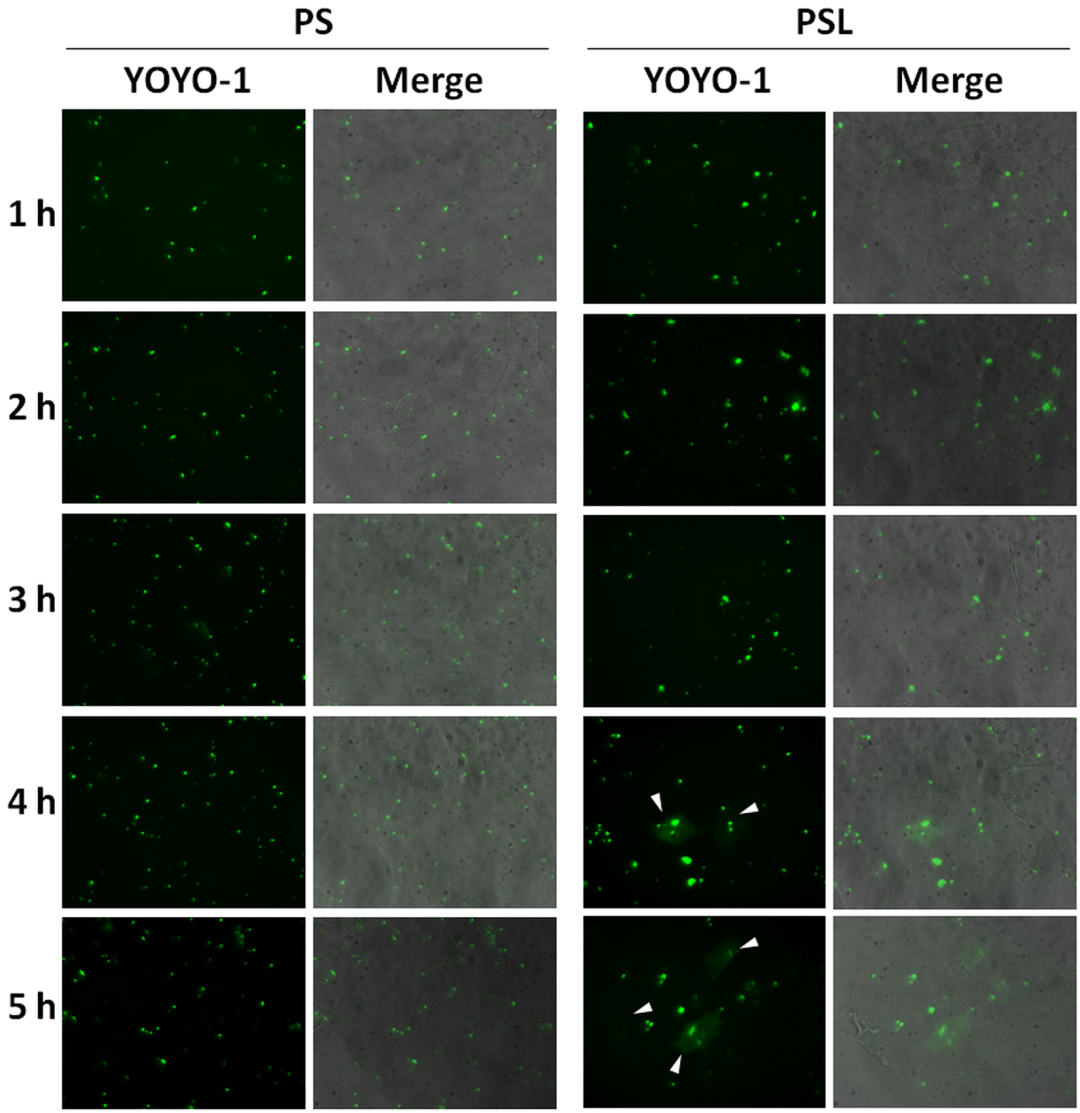
DNA release kinetics of PS and PSL complexes in HeLa cells under fluorescence microscope at a magnification of 400-fold. Plasmid DNA (SB transposase) was labeled with YOYO®-1(green). Arrows show the release of plasmid DNA into the cytosol.

As the SB transposon system contains GFP gene, the expression of GFP gene might disturb the localization of SB transposon system labeled by YOYO^®^-1. To eliminate the interference of GFP signal, PS and PSL complexes without GFP gene were prepared using only SB transposase plasmid. HeLa cells were incubated with PS and PSL complexes without GFP gene for 5 hours. The DNA release kinetics was observed under fluorescence microscope. As shown in Fig. 4, PSL complexes could induce a fast release of plasmid into the cytoplasm. The YOYO^®^-1 signal could be observed in the cytoplasm 4 hours after transfection in the group of PSL complexes. In contrast, PS complexes could not induce sufficient plasmid released into cytoplasm even when the cells were treated with PS complexes for 5 hours. This result confirmed the conclusion that association of LAH4-L1 peptide could facilitate the endosomal escape of PSL complexes, thus resulting in high transfection efficiency.

### Long-term gene expression

Compared to viral gene vectors, non-viral gene vectors have insufficient chromosomal integration activity thus poor stable transfection. In this study, aiming to achieve efficient long-term gene expression using non-viral gene vectors, the sleeping beauty transposon system (SBTS) was chosen as the genetic payload for PSL complexes. SBTS is a plasmid system with the capability of efficient integration of exogenous genes into the host genome, thus leading to a long-term gene expression.

**Figure 5:**
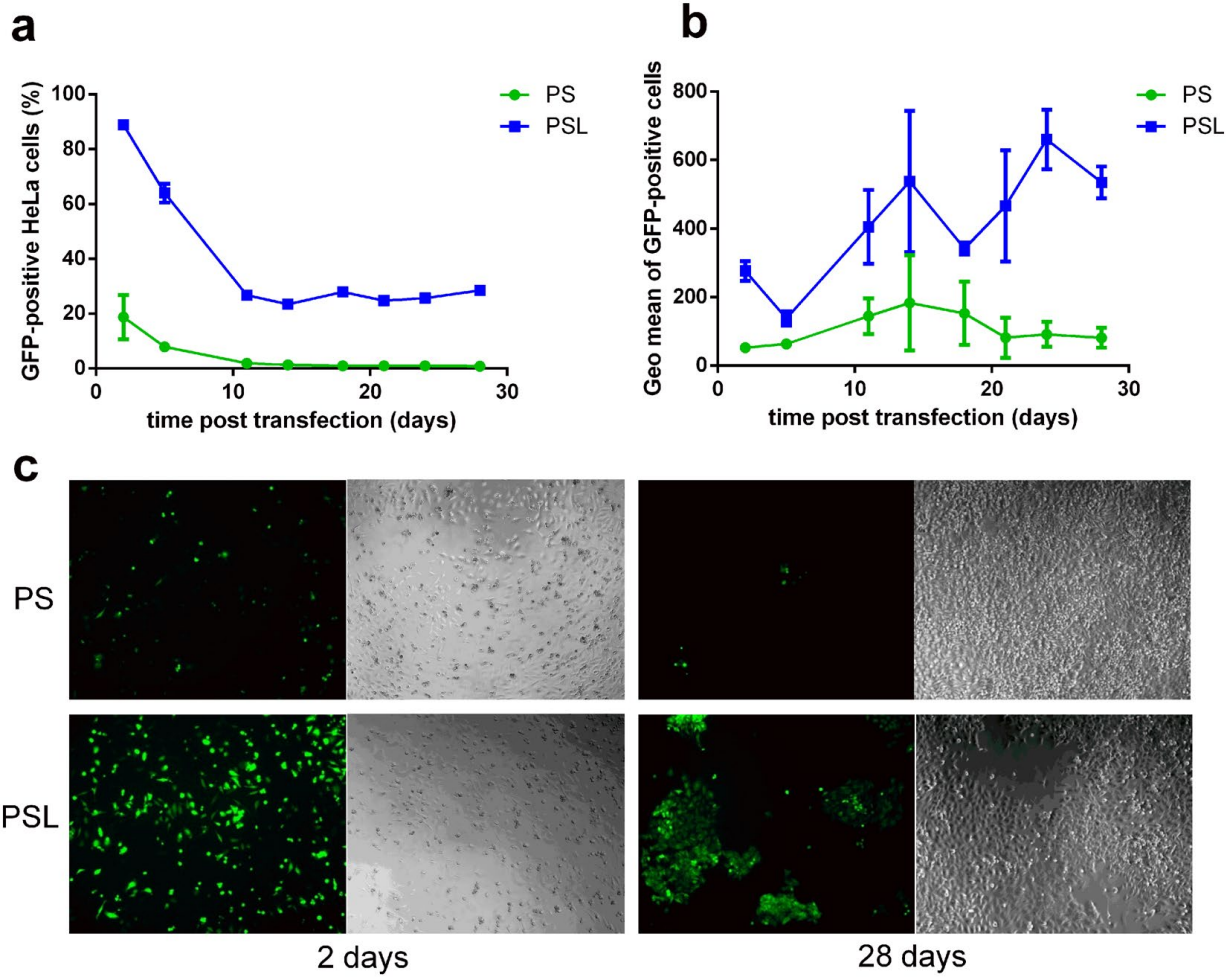
Comparison of long-term gene expression on HeLa cells transfected by PS complexes and PSL complexes in the presence of 10% FCS. Transfection was performed at a dose of 0.3 μg of DNA per well in a total volume of 200 μl. Percentage of GFP-positive cells (**a**) and geometric mean of fluorescence intensity of GFP-positive cells (**b**) were determined by flow cytometry over 28 days post transfection. (**c**) GFP expression visualized under fluorescence microscope 2 days and 28 days post transduction at a magnification of 100-fold. Data represent mean ± standard deviation of 3 independent experiments.

As shown in Fig. 5a,b, PSL complexes could achieve 90% transfection efficiency 2 days post transfection, however, PS complexes could only induce 20% transfection efficiency. 14 days post transfection, PSL complexes could induce 30% transfection efficiency, whereas transfection efficiency of PS complexes dropped to less than 1%. 28 days post transfection, PSL complexes could still achieve 30% transfection efficiency.

The results of flow cytometry were also corroborated by fluorescence microscopy. The GFP expression of HeLa cells was imaged under fluorescence microscope 2 days and 28 days post transfection. As shown in Fig. 5c, a significant number of GFP cell clones were observed when cells were transfected by PSL complexes. In contrast, only a few GFP cells could be detected in the PS group.

### Transfection and cellular uptake of PS and PSL complexes in the presence of serum

**Figure 6:**
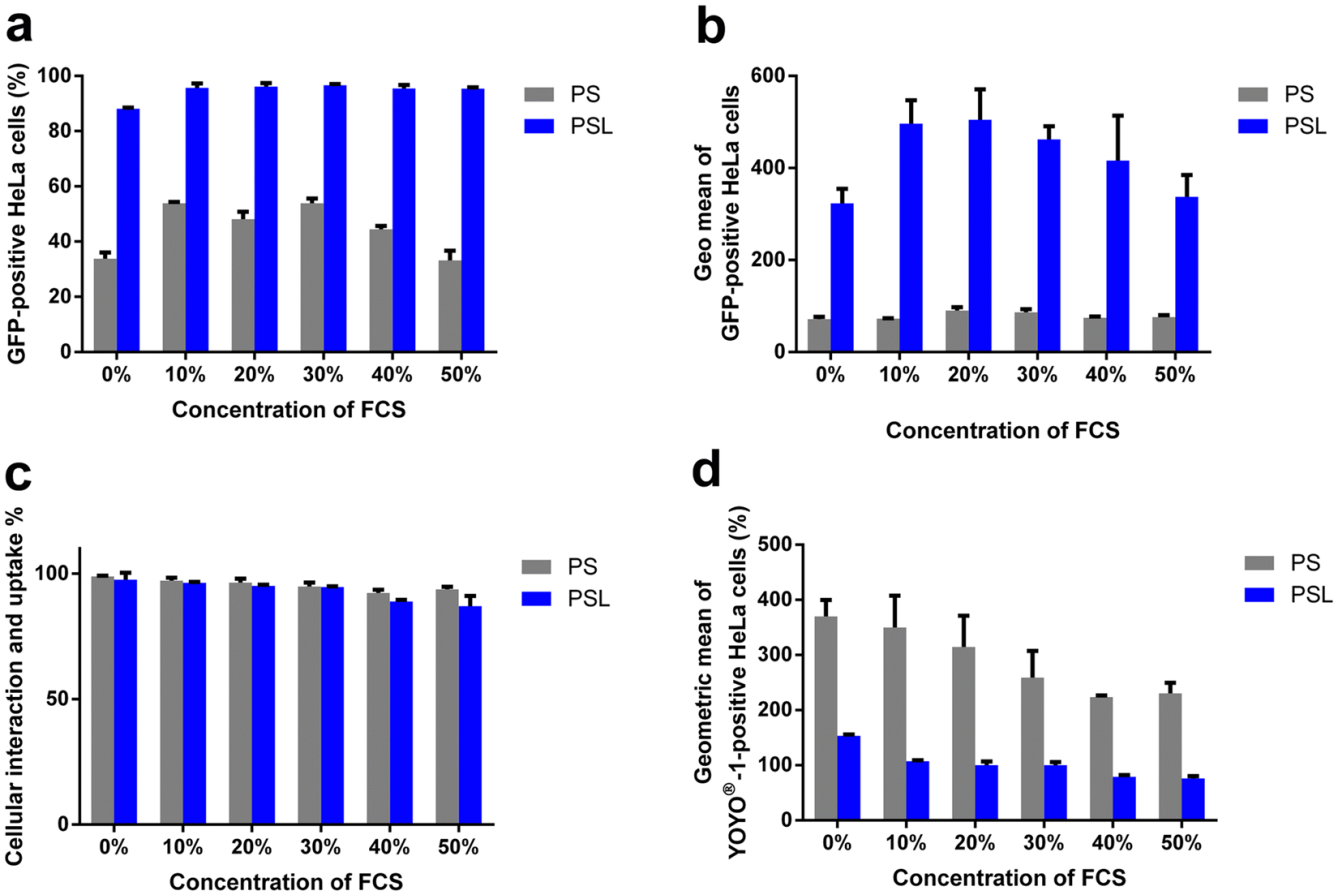
Transfection and cellular uptake of PS and PSL complexes in fetal calf serum (FCS) conditions. Transfection and cellular uptake studies were performed at a dose of 0.3 μg of DNA per well of 48-well plate. (**a**) Effect of serum on the transfection efficiency of PS and PSL complexes. Transfection was performed at various serum concentrations. (**b**) Mean fluorescence intensity of GFP-positive HeLa cells was measured by flow cytometry 24 h after transfection at various serum concentrations. (**c**) Cellular uptake of PS and PSL complexes as a function of serum concentrations. (**d**) Geometric mean of fluorescence intensity (Geo mean) of fluorescent cells as a function of serum concentration. Data represent mean ± standard deviation of 3 independent experiments.

In order to investigate the anti-serum ability of PSL complexes, transfection was performed in the presence of 0%, 10%, 20%, 30%, 40% and 50% FCS, respectively. Figure 6a shows that PSL complexes could induce higher transfection efficiency in the presence of serum than in serum-free medium. Moreover, the percentage of GFP-positive cells transfected with PSL complexes was not impaired in the presence of high concentrations of serum. As shown in Fig. 6b, only the geometric mean of GFP-positive cells was slightly decreased with the increase of serum concentration over 20% in the group of PSL complexes, but it was still over 3-fold than that in the group of PS complexes.

Enhanced transfection efficiency of gene vectors is generally contributed to the improved cellular uptake^21^. Therefore, the difference between the cellular uptake of PS and PSL complexes in the presence of serum was determined by flow cytometry. As shown in Fig. 6c,d, the percentage of cellular uptake was efficient for both PS and PSL complexes in the presence of serum. Even with 50% of FCS, both PS and PSL complexes could be internalized by over 90% of cells. However, the geometric mean of YOYO^®^-1-positive cells decreased with the increase of serum concentration, suggesting that fewer complexes were internalized into cells at higher concentration of FCS. Surprisingly, the mean fluorescence intensity of PSL complexes was significantly lower than that of the PS complexes in all investigated serum concentrations.

The cellular uptake study also confirmed, that other factors, such as endosomal escape rather than enhanced cellular uptake are the crucial reasons for enhanced transfection mediated by PSL complexes.

Overall, among the serum concentration of 10% to 50%, PSL complexes showed efficient transfection efficiency compared to PS complexes, suggesting that PAMAM-mediated gene transfection in the presence of serum was significantly improved by the association of LAH4-L1 peptide.

### Transfection in other cell lines

It has been shown that the transfection efficiency is cell type dependent. In order to further substantiate the enhanced transfection efficiency of PSL complexes in the presence of serum, transfection was performed in different cell lines, including the hard-to-transfect MDCK cell line in the presence of 10% and 50% FCS.

**Figure 7:**
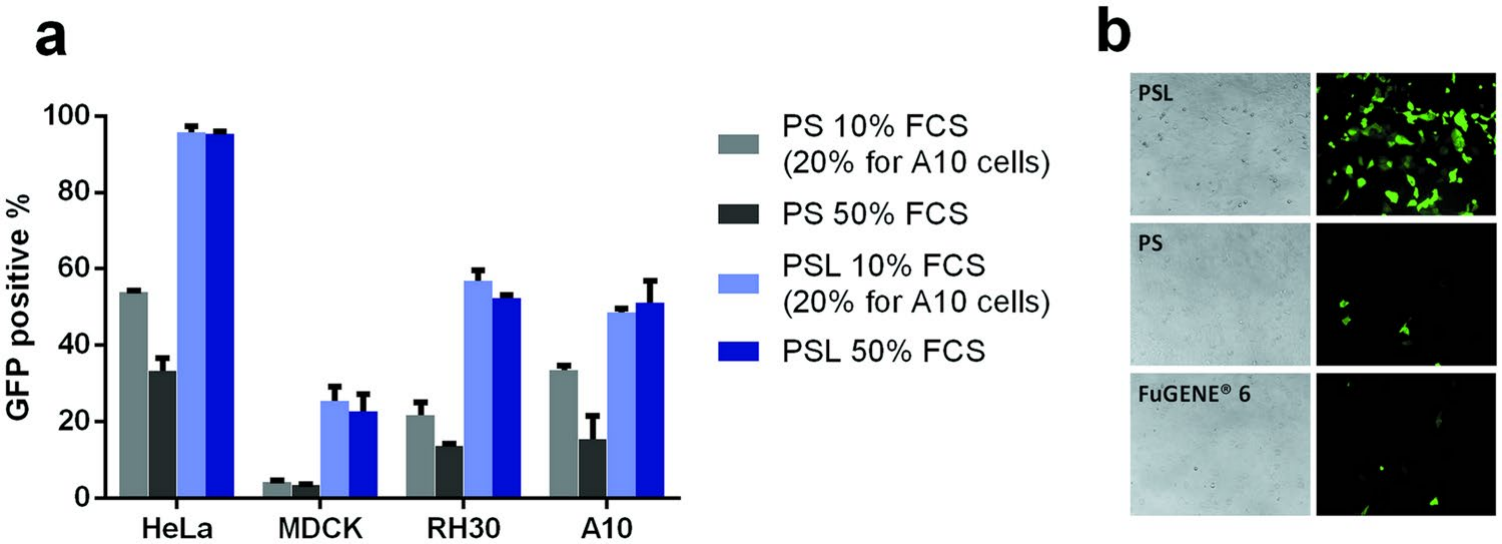
Transfection of other cell lines. (**a**) Comparison of other cell lines transfected with PS and PSL complexes in different concentrations of FCS. Transfection efficiency determined by flow cytometry. (**b**) Comparison of PSL complexes mediated transfection with FuGENE® HD and FuGENE® 6 in MDCK cells. Transfection was performed at a dose of 0.3 μg of DNA per well in a total volume of 200 μl. GFP expression was quantified by fluorescence microscope. Data represent mean ± standard deviation of 3 independent experiments.

As shown in Fig. 7a, among all cell lines, PSL complexes induced higher transfection efficiency in medium containing 10% and 50% of FCS compared to PS complexes. In addition, increasing the serum concentration did not affect the transfection efficiency of PSL complexes. In contrast, GFP expression of PS complexes decreased with the increase of serum concentration. Especially in hard-to-transfect cell lines such as MDCK cells^22^, PSL complexes could achieve improved transfection efficiency compared to PS complexes in the presence of serum. As shown in Fig. 7b, the number of GFP cells was higher when MDCK cells were transfected by PSL complexes. In contrast, only a few GFP cells could be observed in PS group and FuGENE^®^6 group. The transfection efficiency of PSL complexes is further confirmed in Jurkat cells and HEK 293 cells. As shown in Supplementary Figure 5, PSL complexes induced higher transfection efficiency compared to FuGENE^®^6 or Lipofectamine LTX. As compared with PAMAM G-5 based transfection reagent, PSL exhibited higher efficiency compared to Superfect and comparable transfection efficiency to TransExcellent in HEK 293 cells and HeLa cells.

## Discussion

Non-viral vectors, as alternatives to viral vectors, are widely used and investigated for the delivery of genetic material into cells. The majority of non-viral vectors are cationic vectors, which deliver nucleic acids by forming cationic complexes via electrostatic interactions^23^. This basic mechanism is beneficial for nucleic acids packing and protection, cellular uptake and “proton sponge effect” mediated endosomal escape to some extent^1^. However, It has been also reported that the “proton sponge effect” is not sufficiently effective for endosomal escape of DNA from cationic polymer based gene vectors. After successful internalization, an average of only one/two endo/lysosomes burst by the “proton sponge effect”^24^.

To escape from the endosome, PAMAM-mediated transfection requires a high N/P ratio to offer sufficient buffer capacity to trigger endosomal swelling and burst, however, the presence of excessive PAMAM can cause significant cytotoxicity due to the damage of cellular membranes. In contrast, at low N/P ratio, although PAMAM-mediated transfection shows lower cytotoxicity, it results in low transfection efficiency owing to insufficient endosomal release. Despite the low cytotoxicity of the SL complexes, the transfection is very low when cells were transfected by SL complexes. In contrast, PSL complexes could overcome this dilemma by inducing extremely high transfection efficiency in HeLa cells at a low N/P ratio without cytotoxicity. Many cationic non-viral gene vectors are toxic *in vivo*. Specifically, they result in hemolysis due to their high-positively charged surfaces which facilitate the binding of gene vector onto the cell surface. The hemolysis assay (Supplementary Figures 6 and 7) showed that PSL complexes results in very low-level of hemolysis; however, the PS complexes prepared at high N/P raito, free PAMAM or free LAH4-L1 peptide cause aggregation of erythrocytes and hemolysis to some extent. By forming PSL complexes at the low N/P ratio, minimal amount of PAMAM dendrimer and LAH4-L1 is required to form complexes for efficient gene delivery. Thus, the hemolysis caused by non-viral gene vectors can be reduced.

**Figure 8:**
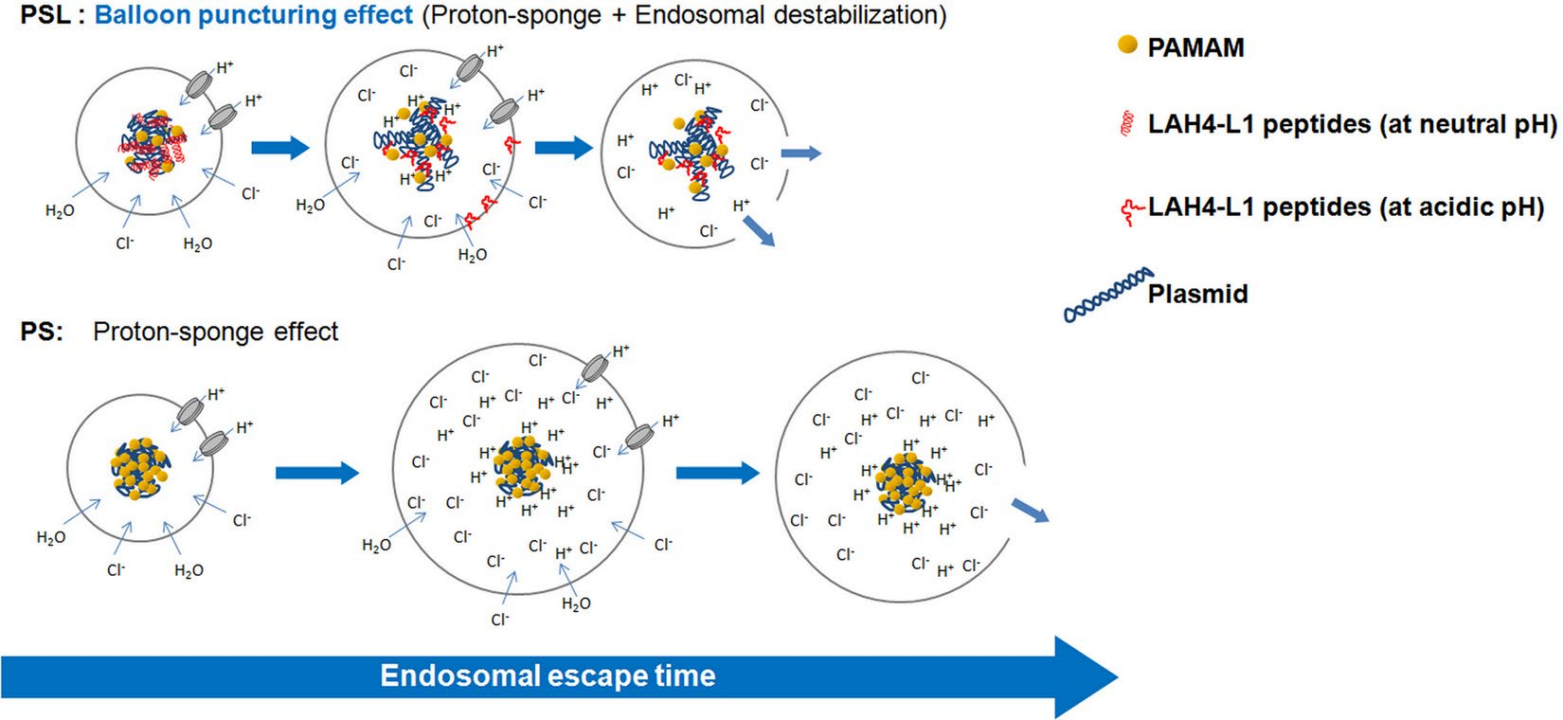
Hypothesis of “Balloon puncturing effect”. PSL complexes possess two endosomal escape strategies for DNA including the proton sponge effect and an additional endosomal destabilization effect, thus resulting in a synergistic and much more efficient ability of endosomal escape. More DNA from PSL complexes can escape from endosomes in lesser time. In contrast, endosomal escape of DNA from PS complexes is only triggered by the proton sponge effect that requires swelling and rupture of the endosomal membrane, which has been proven to be less effective.

For PSL complexes, a new endosomal escape strategy (endosomal destabilisation) was added by introducing the LAH4-L1 peptide to PAMAM based gene delivery vectors. LAH4-L1 peptides are released from the PSL complexes duo to the conformational change when the four histidine residues are positively charged at low pH and enable destabilization of the endosomal membrane^12, 14^. Meanwhile, PAMAM dendrimer triggers the endosomal swelling and burst by the proton sponge effect. Besides, the release of LAH4-L1 peptides from the PSL complexes leads to the disassociation of PSL complexes, which might have a beneficial effect on releasing the DNA into the cytosol. Thus, PSL complexes possess a more efficient endosomal escape strategy combining both the proton sponge effect and the endosomal destabilization effect, called “balloon puncturing effect” (Fig. 8). To confirm this hypothesis, PAMAM was replaced by Polyethyleneimine (PEI), and the transfection efficiency of PEI-mediated gene delivery systems was enhanced by incorporation of LAH4-L1 peptide in the cationic polymer based gene vectors (Supplementary Figure 8).

Long-term therapeutic gene expression is desirable in a variety of gene therapy clinical trials^25^. Compared to viral vectors, non-viral vectors are often less effective for long-term gene expression because of the inefficient integration of single genes into the chromosomes. In this study, the sleeping beauty transposon system with GFP as reporter gene was used for transfection, which performs efficient integration of gene into the host genome and thus provides long-term expression of the gene of interest in cells. Our results demonstrated that PSL could induce long-term GFP-expression up to 28 days in HeLa cells with high efficiency, thus providing a promising and efficient method for long-term gene expression using a non-viral method.

The clinical applications of gene vectors *in vivo* require efficient transfection in the presence of serum. However, high concentration of serum is known to affect the efficiency of cationic non-viral gene vectors due to their unspecific binding to serum protein and inefficient cellular uptake^26, 27^. LAH4-L1 peptide has been proven to have high capability of serum resistance for DNA and siRNA delivery. The transfection results show that adding of LAH4-L1 peptide to PAMAM vectors could significantly enhance its anti-serum ability in all investigated cell lines.

## Conclusions

In this study, we have demonstrated an attractive and efficient strategy to overcome the efficiency-cytotoxicity dilemma of cationic polymer-mediated transfection, enhancing the ability of endosomal escape by the association of LAH4-L1 peptides to PAMAM/DNA complexes via electrostatic self-assembly. LAH4-L1 peptides markedly improved PAMAM-mediated transfection at low N/P ratios in the presence of serum in several cell lines, including hard-to-transfect cell lines. Furthermore, PSL complexes induced long-term gene expression by using SBTS as its genetic payload in HeLa cells. The visualization of intracellular trafficking indicated that LAH4-L1 peptide could improve the release of plasmid DNA into the cytosol. Taken together, this study demonstrates that the association of LAH4-L1 peptides with PAMAM/DNA complexes is a promising strategy for efficient gene delivery.

## Materials and Methods

### Materials

LAH4-L1 peptide was supplied by Burkhard Bechinger (University of Strasbourg/CNRS, Strasbourg, France). PAMAM dendrimer (generation 5, ethyleneadiamine core) was supplied by Yunjun Luo (Beijing Institute of Technology, Beijing, China). Linear polyethylenimine (PEI), 25 kDa was purchased from Polyscience (Warrington, PA). The SB transposon system (pcGlobin2-SB100Xco plasmid: 6640 bp; SB-amaxaGFP plasmid: 5611 bp) was a gift from Toni Cathomen (Institute for Cell and Gene Therapy, University Medical Center Freiburg, Freiburg, Germany). YOYO^®^-1 Iodide and Lipofectamine LTX were obtained from Life technologies, Thermo Fisher Scientific Inc. FuGENE^®^6 was obtained from Promega. Superfect was obtained from QIAGEN. TransExcellent was obtained from Shagnhai Cenji Biotech Co., LTD. All other reagents were obtained from Sigma-Aldrich (St Louis, MO).

### Cell culture

MDCK (an epithelial Madin-Darby canine kidney cell line, ATCC), HeLa (a human cervical carcinoma cell line, ATCC) cells were cultured in Dulbecco’s modified Eagle’s medium (DMEM) containing 10% (v/v) FCS. RH-30 (a Human Rhabdomyosarcoma Cell Line, ATCC) cells and Jurkat (a human T lymphocyte cell line, ATCC) cells were cultured in RPMI 1640 medium containing 10% (v/v) FCS. A10 (vascular smooth muscle cell line, ATCC) cells were cultured in DMEM containing 20% (v/v) FCS. HEK 293 (a human embryonic kidney cell line, ATCC) cells were cultured in DMEM containing 10% (v/v) FCS. All cells were maintained at 37 °C in a humidified incubator with 5% CO_2_ atmosphere.

### Plasmid preparation

SBTS containing GFP gene was amplified in *E. coli* (strain DH5α) in LB medium containing ampicillin, and then isolated and purified using an endotoxin-free plasmid Giga Kit (Qiagen) according to the manufacturer’s instructions. The plasmid was purified with ice-cold 70% ethanol. The concentration and purity of the plasmid were assessed using a spectrometer at 260 nm and 280 nm. The solution of transposon plasmid (SB-amaxaGFP) and transposase plasmid (pcGlobin2-SB100Xco) was diluted and a mixture of plasmids was prepared at the weight ratio of transposon plasmid to transposase plasmid at 3. The final concentration of this plasmid mixture is 1 µg/µl. The plasmid mixture was aliquoted into 0.5 ml microcentrifuge tubes and stored at −20 °C for further use.

### Preparation of PS complexes and PSL complexes

SB/LAH4-L1 complexes at various N/P ratios were prepared by vortexing SBTS with LAH4-L1 peptide in DMEM for 3 seconds at 2500 rpm. The mixture was then incubated at 37 °C in a thermomixer (Comfort, Eppendorf) for 20 min at 300 rpm to form SB/LAH4-L1 complexes. The formed SB/LAH4-L1 complexes were subsequently added into the PAMAM solution, followed by incubation at 37 °C in a thermomixer (Comfort, Eppendorf) for 20 min at 300 rpm to form PSL complexes. PS complexes at various N/P ratios were prepared by vortexing SBTS with PAMAM dendrimers in DMEM for 3 seconds at 2500 rpm. The mixture was then incubated at 37 °C in a thermomixer (Comfort, Eppendorf) for 20 min at 300 rpm to form PS complexes.

N/P ratios were calculated based on the number of terminal amine groups within a PAMAM dendrimer and the number of phosphate groups of the plasmid DNA. In this study, the weight ratio of PAMAM to DNA is 6.8 when the N/P ratio is 10.

### Characterization of SL complexes, PS complexes and PSL complexes

The hydrodynamic diameters and zeta-potential of the PSL, PS and SL complexes were determined by using a Malvern Zetasizer (Malvern, UK). Prior to the measurement, the PSL, PS and SL complexes were prepared at indicated N/P ratios in deionized water. In these measurements, the concentration of SB transposon system was 5 μg/ml. The samples were transferred into the 1.5 ml disposable cuvettes and pre-warmed to 25 °C before the measurement.

Agarose gel electrophoresis was performed at 110 V for 30 min. After electrophoresis, the gel was transferred into the GelGreen^TM^ solution for staining. The location of DNA was visualized with UV irradiation.

### Transfection studies

Cells were seeded into 48-well culture plates 24 hours prior to the experiment at a density of 2 × 10^4^ cells per well and maintained at 37 °C in a humidified incubator with 5% CO_2_ atmosphere. The culture medium was replaced with 200 µl fresh culture medium containing 10% FCS 1 hour prior to transfection (20% FCS for A10 cells). The cells were subsequently incubated with PS complexes, SL complexes and PSL complexes for 4 hours. After incubation, the medium was replaced with fresh culture medium and the GFP expression was analyzed by flow cytometry 24 hours after transfection.

For long-term gene expression, cells were seeded and transfected as described above. The GFP expression was analyzed by flow cytometry and fluorescence microscopy over 28 days after transfection.

For transfection in the presence of serum, cells were seeded as described above. The culture medium was replaced with 200 µl fresh culture medium containing the indicated concentrations of FCS, ranging from 0% to 50%, 1 hour prior to transfection. The cells were subsequently incubated with PSL, PS and SL complexes for 4 hours. After incubation, the medium was replaced with fresh culture medium and the GFP expression was analyzed by flow cytometry or fluorescence microscope 24 hours after transfection.

For transfection using commercial transfection reagents, transfections and media changes were performed according to the manufacturers’ recommendations.

### Analysis by flow cytometry

24 hours post transfection, culture medium was removed and the cells were washed with 0.5 ml PBS without Ca^2+^/Mg^2+^ once for HeLa cells and RH30 cells, twice for MDCK and A10 cells. Cells were harvested by trypsinization with 0.1 ml trypsin/EDTA for 3 minutes. All treated cells were collected by centrifugation at 4 °C for 4 min at 260 g. After centrifugation, cells were washed with PBS twice and resuspended in 200 µl PBS containing Ca^2+^/Mg^2+^. Samples were kept cool on ice in the dark until analyzed. 10,000 events were analyzed from each sample by the BD FACSCalibur^®^ (Becton-Dickinson, Heidelberg, Germany). The transfection efficiency was calculated using CellQuest Pro.

### Cell viability assay and apoptosis assay

Cell viability was determined by CellTiter-Glo^®^ luminescent cell viability assay according to the manufacturer’s protocol. Briefly, after transfection procedure, the 96-well plate and its contents were equilibrated at room temperature for 30 minutes. CellTiter-Glo^®^ reagent was thawed and 100 µl of the CellTiter-Glo^®^ reagent were added to 100 µl of medium containing cells in each well. The plate was shaken on a microplate shaker at 600 rpm for 2 minutes to induce cell lysis. The plate was then incubated at room temperature for 10 minutes. 100 µl sample of each well were transferred into a luminometer-compatible multiwell plate and luminescence of the sample was recorded by a luminometer (MicroLumat Plus LB 96 V Luminometer, Bad Wildbach, Germany).

Apoptosis assay was performed by using the apoptosis assay kit (ThermoFisher). Briefly, after transfection procedure, cells were collected and washed with PBS twice. Subsequently, cells were resuspended with 100 µl of binding buffer. 5 µl of Annexin-V-FITC solution and 5 µl of PI solution were added into the cell suspension. After incubation at room temperature for 10 minutes, 400 µl of binding buffer were added into the cell suspension and 10,000 events were analyzed from each sample by the BD FACSCalibur^®^. The apoptosis and necrosis were calculated using CellQuest Pro.

### Hemolysis assay

*In vivo* toxicity of the PS complexes, SL complexes and PSL complexes was evaluated by the hemolysis assay. Blood from C57BL/6 mouse was anticoagulated with EDTA. Erythrocytes were separated from the whole blood by centrifugation at 1000 g for 10 min at 4 °C and washed three times with PBS. Erythrocytes at a hematocrit of 2% were suspended in PBS and incubated with different HSA-dendriplex solutions at concentration of 4.5 mg/mL and incubated with PS complexes, SL complexes and PSL complexes, free PAMAM or free LAH4-L1 peptide at room temperature for 4 hours.

For microscopy, samples were viewed after incubated at 37 °C for 3 h using a fluorescence microscope with a 40x objective (Olympus IX 73). To quantify the level of hemolysis, samples were centrifuged at 1000 g for 10 minutes and the absorbance of each supernatant was measured at 540 nm.

### Cellular uptake study

SBTS was labelled with YOYO^®^-1 at a staining ratio of 1 YOYO^®^-1 per 60 base pairs for 10 minutes before preparation of PS and PSL complexes. The YOYO^®^-1-labeled complexes were then prepared as described above.

For cellular uptake in the presence of serum, cells were seeded as described before. The culture medium was replaced 1 hour prior to transfection with 200 µl fresh culture medium containing the indicated concentrations of FCS, ranging from 0% to 50%. The YOYO^®^-1-labeled complexes were then incubated with HeLa cells for 4 hours or certain incubation time. After incubation, the cells were harvested and analyzed by flow cytometry.

For the fluorescence microscopy studies, 24 h prior to the experiment cells were seeded onto sterilized 12 mm gelatine-coated coverslips in 24-well plates at a density of 2 × 10^4^ cells per well. The culture medium containing Lysotracker^®^ Red (50 nM) was renewed 1 h prior to incubation and HeLa cells were then incubated with the YOYO^®^-1-labeled complexes for 4 h at 37 °C. Thereafter, cells were washed three times with warm PBS w/o Ca^2+^/Mg^2+^ at 37 °C. Cells were then fixed using 1 ml of a freshly prepared paraformaldehyde solution (4%) for 20 min at 37 °C. Subsequently, cells were washed again three times with warm PBS w/o Ca^2+^/Mg^2+^. Afterwards, coverslips were embedded with MoBiGLOW^®^ (MoBiTec GmbH, Goettingen, Germany) on a slide. The cells were analyzed using a fluorescence microscope with a 63x oil-immersion objective (Axio observer, Zeiss, Jena, Germany).

### Statistical analysis

Data was analyzed by GraphPad 6 (San Diego, California). The results were expressed as means ± standard deviation.

## Electronic supplementary material

Supplemental information
