# Supplementary material for: The histidine-rich peptide LAH4-L1 strongly promotes PAMAM-mediated transfection at low nitrogen to phosphorus ratios in the presence of serum
Source: Sci Rep. 2017 Aug 29;7:9585. doi: 10.1038/s41598-017-10049-y (PMC5575053; doi:10.1038/s41598-017-10049-y)
Supplement: Supplementary file 1 — Supplemental information [file 41598_2017_10049_MOESM1_ESM.doc]

### Supplemental information

**The histidine-rich peptide LAH4-L1 strongly promotes PAMAM-mediated transfection at low nitrogen to phosphorus ratios in the presence of serum**

Nan Liu^1*^, Burkhard Bechinger^2^, Regine Süss^1*^

1 Institute of Pharmaceutical Sciences, Department of Pharmaceutical Technology and Biopharmacy and Freiburger Materialforschungszentrum (FMF), Albert Ludwig University Freiburg, Sonnenstr. 5, 79104 Freiburg, Germany

2 University of Strasbourg/CNRS, Membrane Biophysics and NMR, Chemistry Institute UMR7177, rue Blaise Pascal 1, 67008 Strasbourg, France

*Correspondence should be addressed to Nan Liu ([liunanemail@126.com](mailto:liunanemail@126.com)) or Regine Süss (regine.suess@pharmazie.uni-freiburg.de)


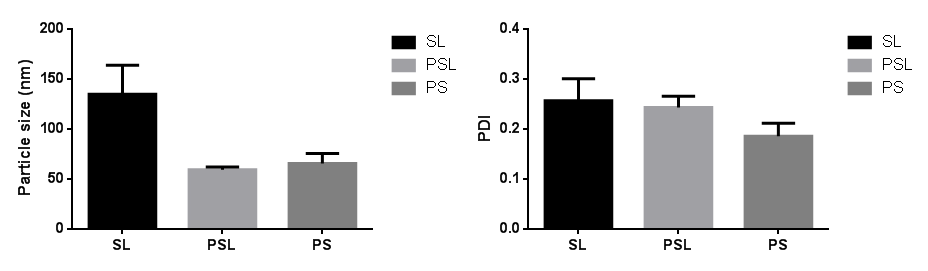


Supplementary Figure 1. Particle sizes of SL, PSL and PS complexes. SL complexes were prepared at a weight ratio of DNA to LAH4-L1 peptide at 1/1. PSL and PS complexes were prepared at N/P ratio of 5 and a weight ratio of DNA to LAH4-L1 peptide at 1/1. Data represent mean ± standard deviation of 3 independent experiments.


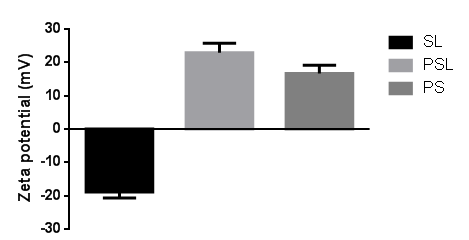


Supplementary Figure 2. Zeta potential measurement of SL, PSL and PS complexes. SL complexes were prepared at a weight ratio of DNA to LAH4-L1 peptide at 1/1. PSL and PS complexes were prepared at the N/P ratio of 5 and a weight ratio of DNA to LAH4-L1 peptide at 1/1. Data represent mean ± standard deviation of 3 independent experiments.


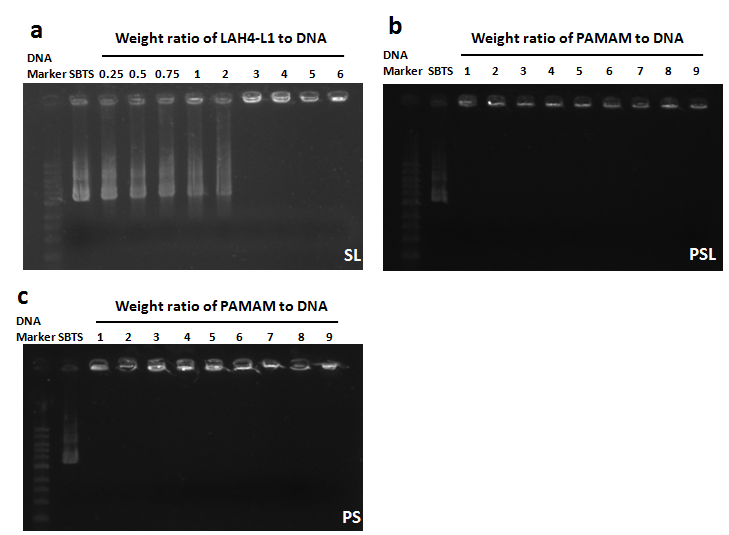


Supplementary Figure 3. Agarose gel retardation assay of SL, PSL and SL complexes. (a) DNA marker (lane 1), naked plasmid DNA (lane 2), SL complexes prepared at various weight ratios of LAH4-L1 to DNA (lane 3-11). (b) DNA marker (lane 1), naked plasmid DNA (lane 2), PSL complexes prepared at various weight ratios of PAMAM to DNA, weight ratio of LAH4-L1 to SBTS was 1 (lane 3-11). (c) DNA marker (lane 1), naked plasmid DNA (lane 2), PS complexes prepared at various weight ratios of PAMAM to DNA (lane 3-11).


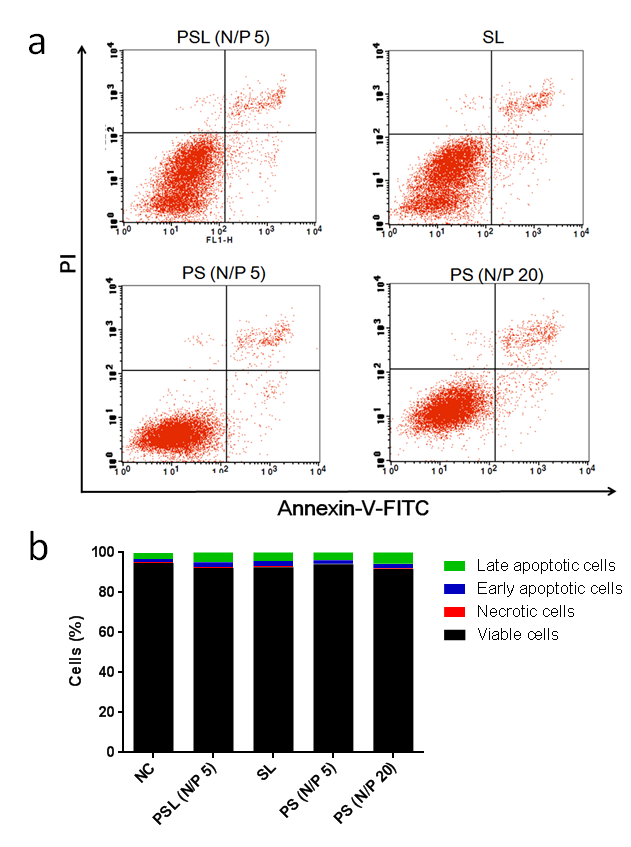


Supplementary Figure 4. Apoptosis assay of SL, PSL and PS complexes. SL complexes were prepared at a weight ratio of DNA to LAH4-L1 peptide at 1/1. PSL complexes were prepared at the N/P ratio of 5 and a weight ratio of DNA to LAH4-L1 peptide at 1/1. PS complexes were prepared at the N/P ratio of 5 or 20 and both PS complexes were prepared at a weight ratio of DNA to LAH4-L1 peptide at 1/1.


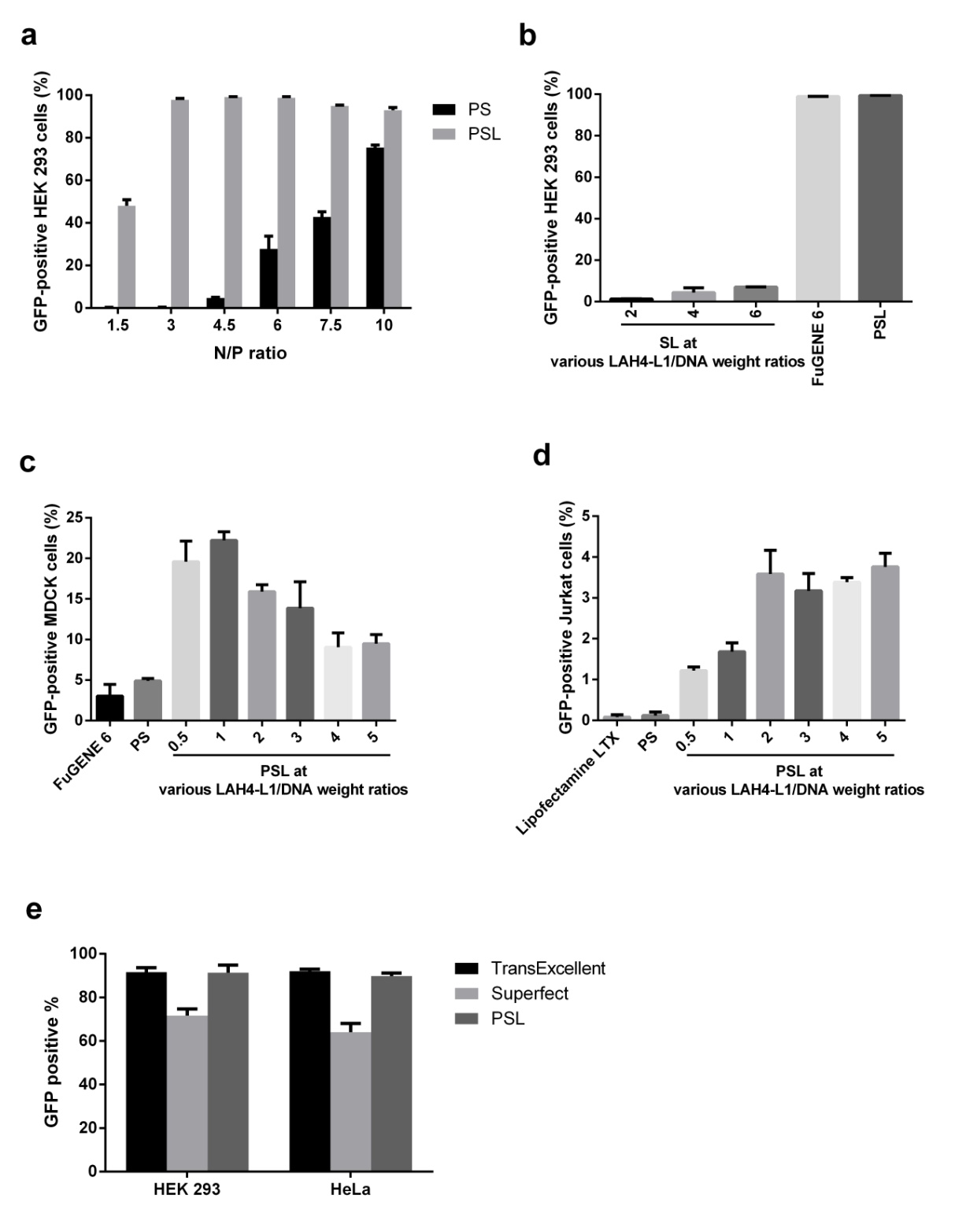


Supplementary Figure 5. (a) Transfection efficiency of PSL complexes and PS complexes in HEK 293 cells at various N/P ratios in the presence of 10% FCS. (b) Transfection efficiency of SL complexes at various LAH4-L1/DNA weight ratios, PSL complexes (N/P ratio of 5 and LAH4-L1/DNA weight ratio of 1) and FuGENE in HEK 293 cells in the presence of 10% FCS. (c) Transfection efficiency of PSL complexes prepared at LAH4-L1/DNA weight ratios range from 0.5 to 5 in MDCK cells. (d) Transfection efficiency of PSL complexes prepared at LAH4-L1/DNA weight ratios range from 0.5 to 5 in Jurkat cells. (e) Transfection efficiency of TransExcellent, Superfect and PSL complexes in HEK 293 and HeLa cells. Data represent mean ± standard deviation of 3 independent experiments.


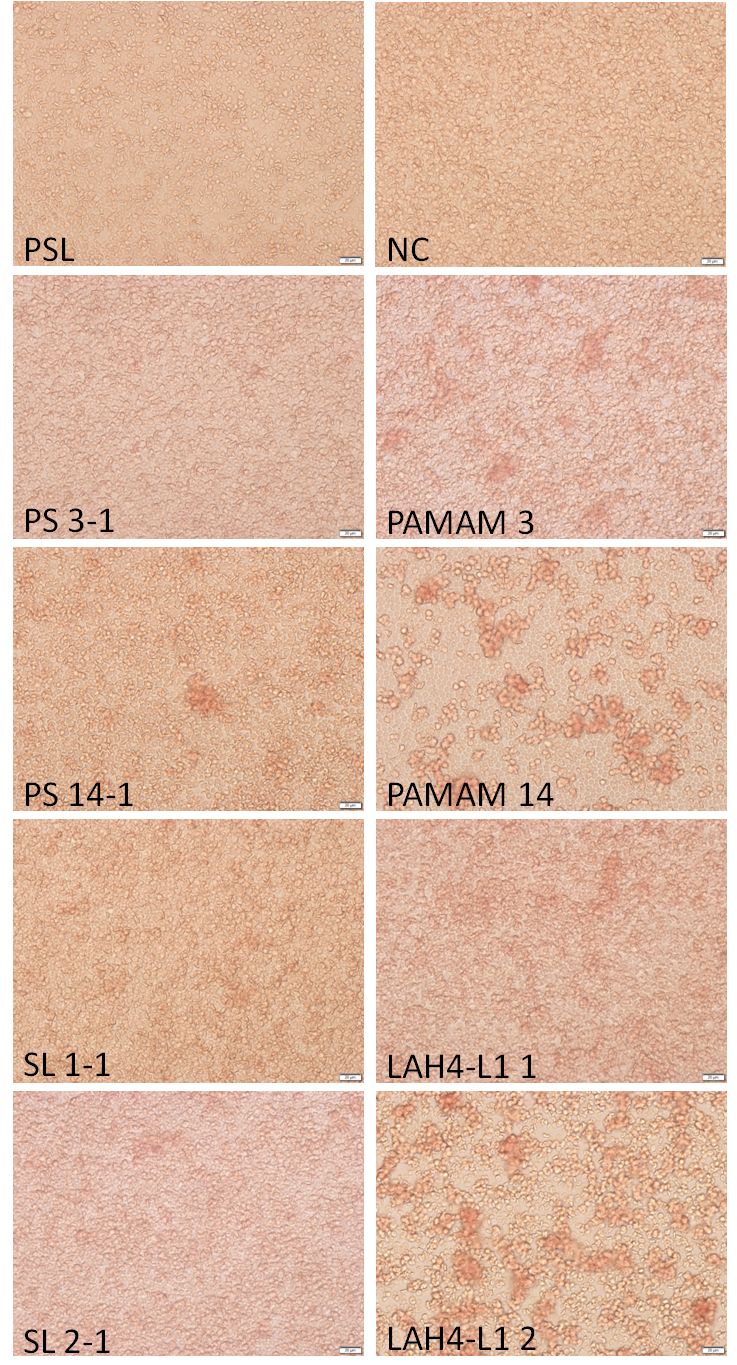


Supplementary Figure 6. Optical micrographs of erythrocytes treated with PSL complexes (PSL: PAMAM/DNA/LAH4-L1, 3/1/1, w/w/w), PS complexes (PS 3-1: PAMAM/DNA, 3/1, w/w; PS 14-1: PAMAM/DNA, 14/1, w/w), SL complexes (SL 1-1: LAH4-L1/DNA, 1/1, w/w; SL 2-1: LAH4-L1/DNA, 2/1, w/w), free PAMAM dendrimers (PAMAM 3: the same amount of PAMAM dendrimer as PS 3-1; PAMAM 14: the same amount of PAMAM dendrimer as PS 14-1) and free LAH4-L1 peptides (LAH4-L1 1: the same amount of LAH4-L1 peptide as SL 1-1; PAMAM 14: the same amount of LAH4-L1 peptide as SL 2-1). Assay was performed at a dose of 0.5 µg of DNA per well in a total volume of 500 µl.


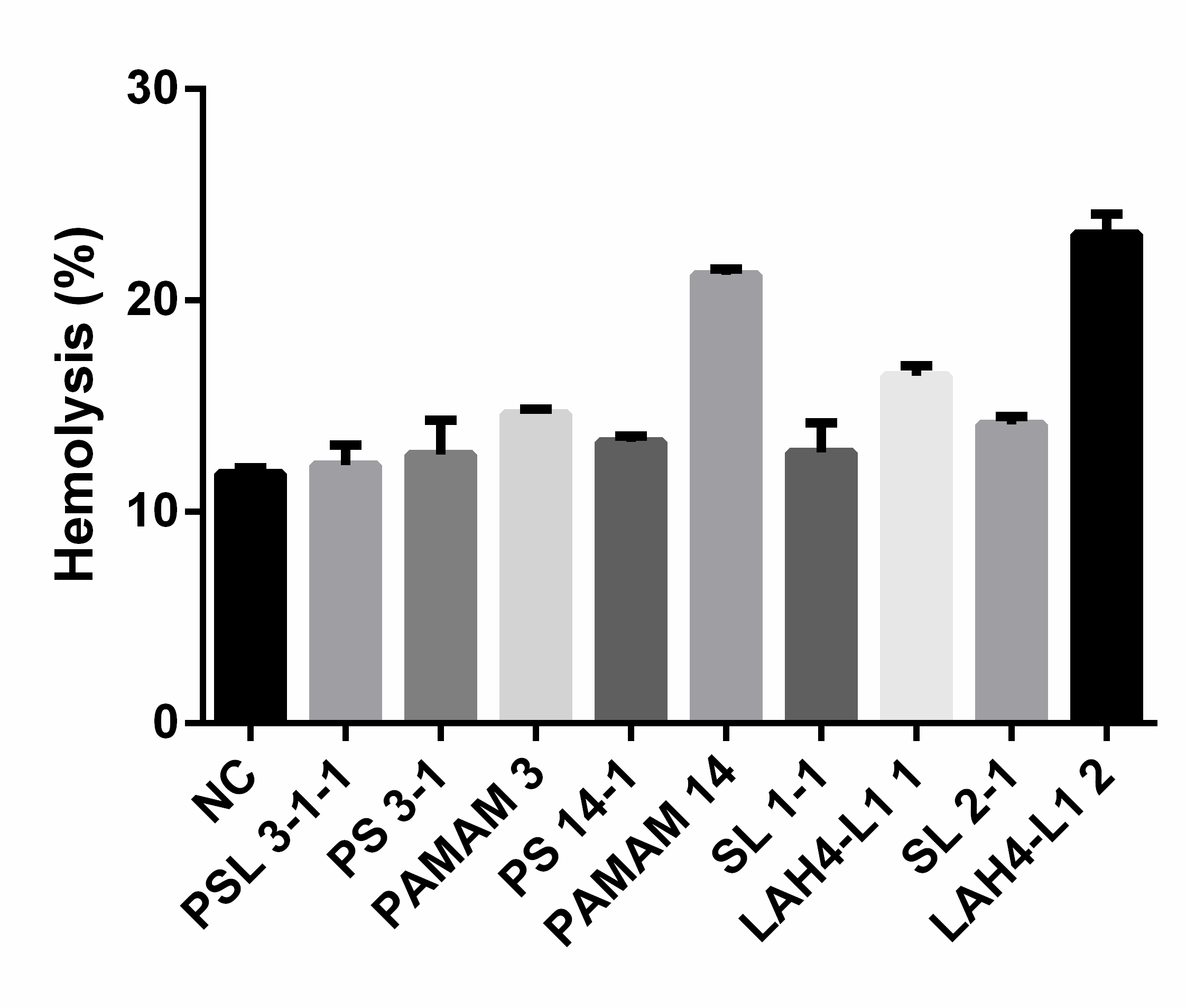


Supplementary Figure 7. Hemolysis assay of erythrocytes treated with PSL complexes (PSL: PAMAM/DNA/LAH4-L1, 3/1/1, w/w/w), PS complexes (PS 3-1: PAMAM/DNA, 3/1, w/w; PS 14-1: PAMAM/DNA, 14/1, w/w), SL complexes (SL 1-1: LAH4-L1/DNA, 1/1, w/w; SL 2-1: LAH4-L1/DNA, 2/1, w/w), free PAMAM dendrimers (PAMAM 3: the same amount of PAMAM dendrimer as PS 3-1; PAMAM 14: the same amount of PAMAM dendrimer as PS 14-1) and free LAH4-L1 peptides (LAH4-L1 1: the same amount of LAH4-L1 peptide as SL 1-1; PAMAM 14: the same amount of LAH4-L1 peptide as SL 2-1). Assay was performed at a dose of 0.5 µg of DNA per well in a total volume of 500 µl.


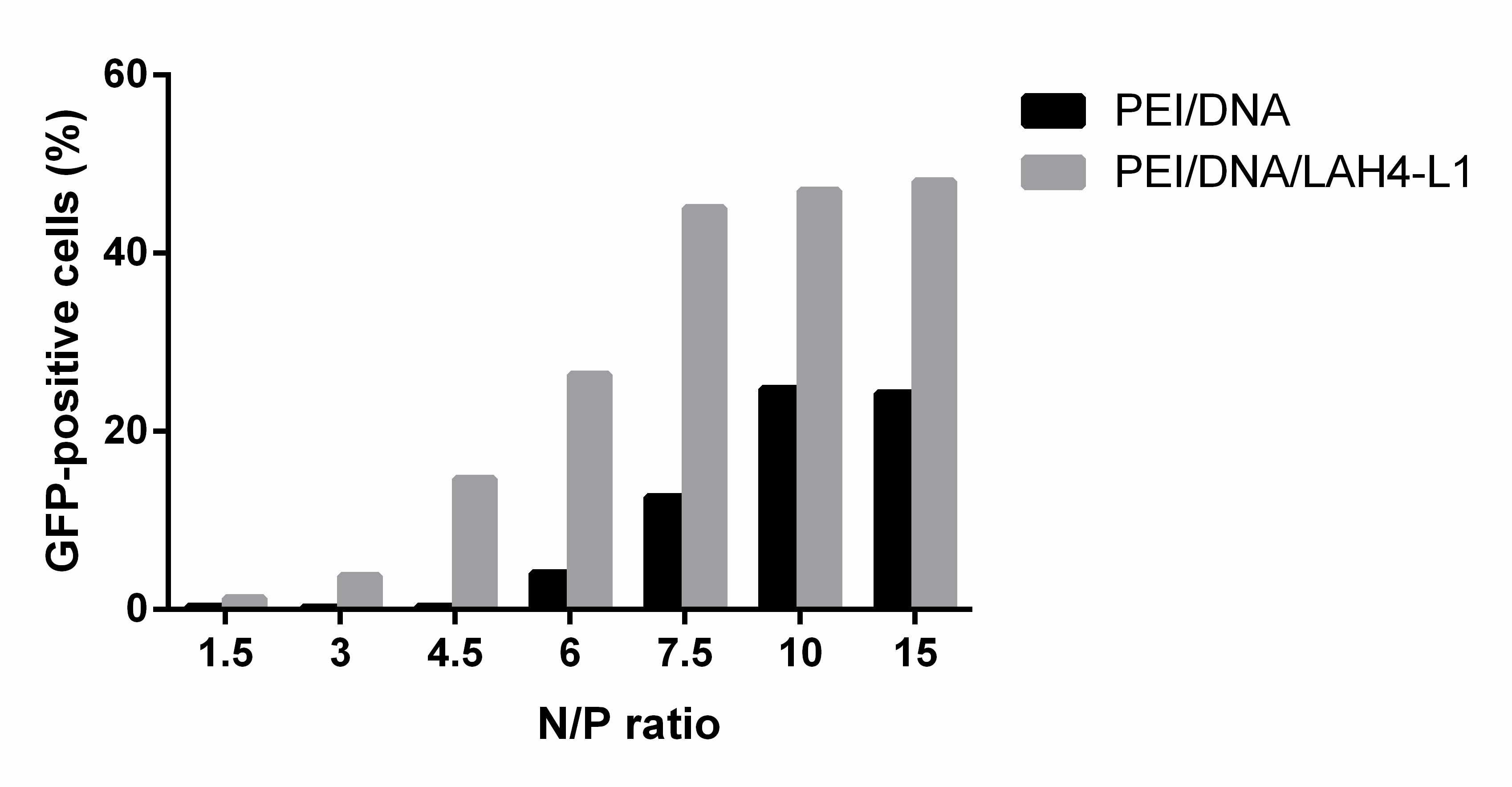


Supplementary Figure 8. Transfection efficiency of PEI/DNA/LAH4-L1 complexes and PEI/DNA complexes in HeLa cells at various N/P ratios in the presence of 10% FCS. PEI/DNA/LAH4-L1 complexes and PEI/DNA complexes were prepared in the same way as preparation of PSL complexes or PS complexes. GFP expression was quantified by flow cytometry after 24 hours.
